# Prevalence and Clinico-pathologic Profile of Biopsied Gingival Lesions from 2 Thai Dental Schools

**DOI:** 10.4317/jced.61932

**Published:** 2024-09-01

**Authors:** Kittipong Dhanuthai, Supissara Boonkhanasan, Panupong Kasarat, Pakkanan Nateetaweewat, Kraisorn Sappayatosok

**Affiliations:** 1 Faculty of Dentistry, Chulalongkorn University; 2 College of Dental Medicine, Rangsit University

## Abstract

**Background:**

Gingiva can be afflicted by several pathological entities apart from gingivitis and periodontitis. There have been limited number of studies on gingival lesions, especially from Thailand. Aim: To analyze the prevalence and distribution of biopsied gingival lesions from Chulalongkorn and Rangsit Dental Schools, Thailand.

**Material and Methods:**

Biopsy records of the participating institutions from 1995 to 2020 were reviewed for gingival lesions. The demographic data, site of lesions and diagnoses were collected. Data were analyzed by descriptive statistics.

**Results:**

From a total of 16,207 biopsies, 1,589 cases (10.2%) were diagnosed in the category of gingival lesions. The mean age ± SD of the patients was 42.3 ± 18.6 years with the highest prevalence being in the fourth decade of life (17.4%). A male-to-female ratio was 0.48:1. The most common location was the posterior mandible (27.4%). Regarding the type of gingival lesions, non-neoplastic lesions (88.5%) outnumbered neoplastic lesions (11.5%). The most common lesion was pyogenic granuloma, followed by irritation fibroma and peripheral ossifying fibroma. Among the neoplastic lesions, squamous cell carcinoma was the most prevalent lesion followed by papilloma and lymphoma.

**Conclusions:**

Gingival lesions mostly occur in the fourth decade of life and have a predilection for female patients. The majority of the lesions are located in the posterior mandible. Non-neoplastic lesion, especially the reactive subtype, is the most prevalent group and pyogenic granuloma is the most common gingival lesion. Data from this study represent biopsied gingival lesions from Thailand which may be different from those of other countries.

** Key words:**Gingival lesions, prevalence, demographic, clinico-pathological correlation, gingival biopsy.

## Introduction

Gingiva is an important part of the periodontium. It acts as a peripheral seal of tooth by junctional epithelium and connective tissues attachment. Gingiva protects underlying periodontal ligament, tooth roots, cementum and alveolar bone from oral pathogen and any trauma in the oral cavity (1). Many oral lesions occur on the gingiva. The etiologies for gingival lesions range from dental plaque induced lesions (2), to non-plaque induced lesions which include neoplastic and non-neoplastic lesions (3). Neoplastic lesion can be further classified as benign and malignant lesions. Non-neoplastic lesions can be reactive lesion, infection, autoimmune, developmental and premalignant lesions. The clinical appearance of gingival lesion can be ulcerative lesion, white lesion, red lesion, lump and bump. Biopsy is the key for diagnosing the gingival lesion because clinical or radiographic finding cannot provide definitive diagnosis. Up until now, there have been limited number of epidemiological studies on gingival lesions (4-15), especially from Asia and Thailand in particular. The objective of this study was to analyze the prevalence and distribution of gingival lesions in a group of patients whose biopsies were sent for the diagnosis at Oral Pathology Department, Faculty of Dentistry, Chulalongkorn University and College of Dental Medicine, Rangsit University and to compare the data with other studies.

## Material and Methods

The study was conducted after approval had been received from the Institutional Review Board of the Faculty of Dentistry, Chulalongkorn University and Rangsit University COA.No. RSUERB2020-048.

A retrospective study on biopsied records from 1,589 gingival lesions from 1st January of 1995 to 31st December of 2021 at the Department of Oral Pathology, Faculty of Dentistry, Chulalongkorn University and College of Dental Medicine, Rangsit university was carried out. Demographic data, site of the lesion, and pathological diagnosis were recorded. The lesions were classified into neoplastic lesions (benign, malignant) and, non-neoplastic lesions (reactive, infection, autoimmune, developmental lesions, premalignant lesions).

The inclusion criteria were the biopsied cases of gingival lesions with complete information regarding demographic data and histopathological diagnoses during the aforementioned period. Bony lesions which produced gingival lumps or bumps were excluded. Gingivitis and periodontitis which are dental plaque induced lesions were also excluded.

The study was approved by ethical committee of both institutions and were in accordance with the ethical standards in the 2008 Declaration of Helsinki and its later amendments. Data were analyzed by descriptive statistics using SPSS version 20.0.

## Results

From a total of 16,207 biopsies, 1,589 cases (10.2%) were diagnosed in the group of gingival lesions. The mean age±SD of the patients was 42.28±18.57 years with the highest prevalence is in the fourth decade of life (17.4%). A total of 1,074 cases (67.6%) were found in women, whereas 515 cases (32.4%) were found in men. A male-to-female ratio was 0.48:1. The most common location was the posterior mandible (27.4%). The frequency of lesions according to disease category is shown in  Table 1.

**Figure 1:**
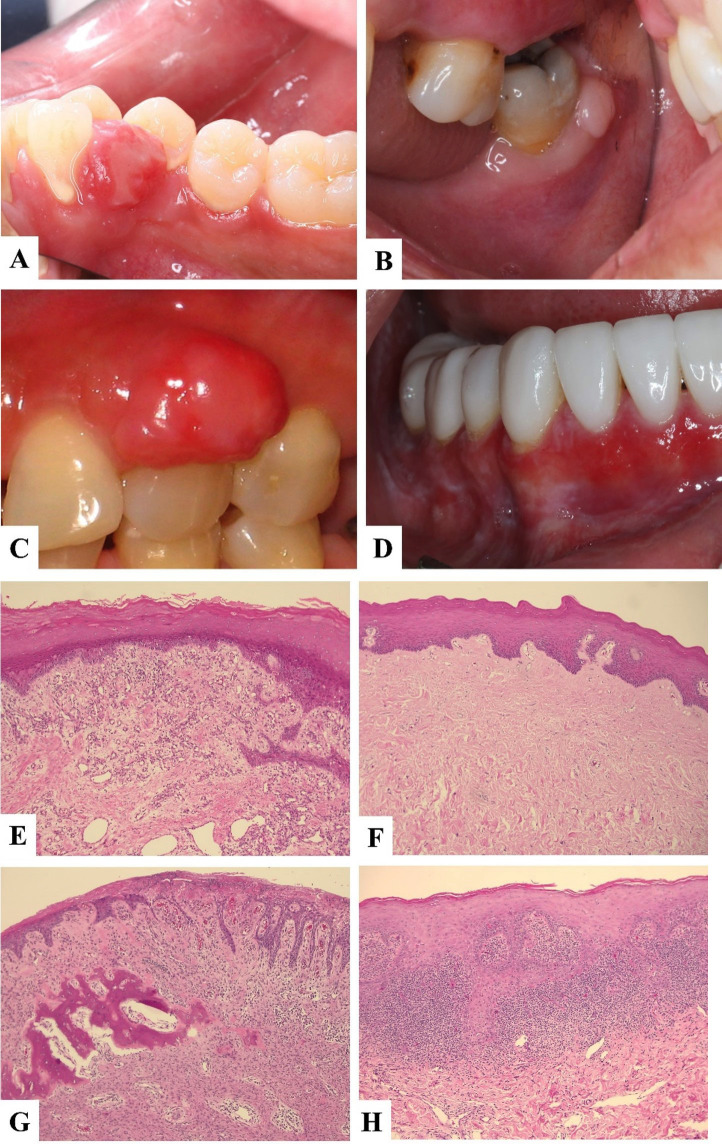
The clinical and histopathological features of four most common gingival lesions; Pyogenic granuloma (A,E), irritation fibroma (B,F), peripheral ossifying fibroma (C,G) and oral lichen planus (D,H).

Regarding the type of gingival lesions, non-neoplastic lesions accounted for the majority of the lesions with the prevalence of (85.3%) which can be subclassified as reactive lesions (69.6%), autoimmune lesions (12.4%), premalignant lesions (1.9%), infection (0.5%) and developmental lesions (1.0%). Neoplastic lesions: malignant lesions (7.8%) and benign lesions (6.9%) constituted 14.7% of the gingival lesions. The most common gingival lesion found was pyogenic granuloma (PG) (30.5%), followed by irritation fibroma (IF) (17.2%), peripheral ossifying fibroma (POF) (9.4%), and lichen planus (LP) (7.7%), respectively. Among the malignancies, oral squamous cell carcinoma (OSCC) was the most frequent malignancy found (47.3%), followed by lymphoma (7.1%) and verrucous carcinoma (VC) (5.5%) respectively. LP is the most prevalent autoimmune lesions (7.7%) followed by mucous membrane pemphigoid (MMP) (2.9%) and pemphigus vulgaris (PV) (1.4%). The clinical and histopathological features of four most common gingival lesions are shown in Figure 1. The distribution of the 10 most commonly found lesions according to patients’ age, gender and site is shown in  Table 2

## Discussion

Gingival lesions range from reactive lesions which are not harmful to detrimental autoimmune and malignant lesions which can be fatal. In our study, gingival lesions constituted 10.2% of all biopsied lesions during the 26-year study period which is comparable to 5.6% by Effiom *et al*. (8), 6.7% by Buchner *et al*. (4), 9.5% Alblowi and Binmadi (5), 18.92% by Montazer Lotf-Elahi (16) while some did not provide the prevalence of gingival lesion in the studies (6-8,10-14).

Mean age of the patients in this study was 42.20 year which is comparable to those of previous studies (4,5,10-14). The majority of gingival lesions in this study were in female (67.60%) which are in accordance with previous studies (4-8,10-14). The peak incidence of lesions was found between the age of 30-39 years which is different from the study by Manjunatha (7) but consistent with study by Alblowi and Binmadi (5). The youngest patient in the study had congenital epulis of newborn, while the oldest was diagnosed with hyperkeratosis.

Most common lesion in this study was non-neoplastic lesion (85.30%) which is in accordance with most studies (4-8,10-12,14) except the study by Li *et al*. (13) which showed that OSCC accounted for 30.53% of gingival lesions. Among the non-neoplastic lesions, most of the studies (5-7) showed that reactive lesions, especially PG was the most commonly found lesion except studies by Gambino (11) which showed higher prevalence of oral potentially malignant disorders than reactive lesions. Study by Hunasgi *et al*. (14) revealed that inflammatory gingival hyperplasia had a higher prevalence than PG and fibrous hyperplasia was shown to have a higher prevalence than PG in the studies by Li *et al*. (13) and Hernandez-Rios *et al*. (12).

The four most common gingival lesions for every disease category in this study were PG, IF, POF and LP. All of them preferentially occurred in female. Study of Hernandez-Rios *et al*. (11) also showed a higher prevalence of fibroma (35.47%) than PG (18.77%), peripheral giant cell granuloma (PGCG) (9.68%) and POF (5.73%).

PG is the reactive lesion which occurs mostly on the gingiva, especially the interdental papilla. In our study, PG afflicted female (71.5%) more than male (28.5%). Common location for PG was the anterior maxilla (28.9%) followed by posterior maxilla (25.2%), posterior mandible (23.1) and anterior mandible (22.7). Mean age of the patient was 38.37 years, with peak incidence at 30-39 age interval (22.1%). The result of the present study is comparable with most studies(4-8,14) except the study of Carbone *et al*. (9) which showed a higher prevalence of fibroma than PG and an equal prevalence of PG and PGCG. The study by Buchner *et al*. (4) showed a higher prevalence of IF than PG.

POF is considered to be a reactive lesion than a neoplasm. The common location for POF in this study was the anterior maxilla (42%) followed by anterior and posterior mandible equally at 20.7% and posterior maxilla (16.7%), respectively. Peak incidence was at 20-29 years (32.7%), with the mean age of 33.21 years. Only the study by Hernandez-Rios *et al*. (12) showed a higher prevalence of PGCG than POF. This discrepancy may be accounted for by the ethnic or genetic background of the patients. However, one study (7) classified POF as a benign neoplasm which makes the inconsistent data through the literature search.

IF was most prevalent in the anterior maxilla (33.0%), anterior mandible (29.3%), posterior mandible (20.1%) and posterior maxilla (17.6%) respectively. Peak incidence was at 20-29 years (22.7%) with mean age of 40.56 years. The result of our study is in accordance with previous studies (3,4,6,11) showing a higher frequency of IF than POF.

LP was the most common autoimmune diseases presenting as desquamative gingivitis in the study, while study by Li *et al*. (12) showed higher prevalence of MMP than OLP. The prevalence of LP in female from this study was 77.9%. Mean age of the patient was 49.70 years with peak incidence at 50-59 years (29.5%). Most lesions occurred at multiple locations within the mouth (88.5%), not only on the gingiva. The percentage of LP manifested only on gingiva in this study (11.5%) is slightly higher than 7.80% by Arduino *et al*. (9) and 7.40% by Fedele *et al*. (15). Among the lesions occurred solely on the gingiva, both mandible and maxilla gingival lesion were the most affected sites (89.3%).

The present study demonstrated that the prevalence of malignant neoplastic lesions (7.8%) outnumbered the benign counterpart (6.9%) which is in agreement with previous studies (10,12,16). Benign gingival lesions accounted for 6.9% which is comparable to the study by Alblowi and Binmadi (5), while some studies (6,7) showed a much higher frequency of benign gingival lesions since they classified IF, POF and peripheral giant cell granuloma as benign neoplastic gingival lesions (7). Clinical appearance of most benign lesions looks the same, usually presenting as non-ulcerated mass on the gum, some of which may resemble reactive lesions, so biopsy is the gold standard for the diagnosis of these lesions. Giant cell fibroma was the most common benign lesion in this study (3.08%), followed by squamous papilloma (2.14%) and lipoma (0.37%), respectively. Study of Carbone *et al*. (9) classified giant cell fibroma as a non-neoplastic disease and showed a frequency of 4.29% of all lesions in the study. Study by Hernandez-Rios *et al*. (11) showed a slightly higher frequency of squamous papilloma than giant cell fibroma (2.17 vs 2.08%), while the study by Li *et al*. (13) showed that verruciform xanthoma and squamous papilloma were the two most prevalent lesions in this group (1.22 vs 1.15%).

In the present study, malignant neoplastic gingival lesions constituted 7.8% of all gingival lesions which is consistent with 2.0-8.0% in previous studies (5-7,11,12). Among these, OSCC was the most prevalent gingival malignant tumor as in previous study (17). Although the prevalence of malignant tumors at the gingiva is low, they do exist and this reiterates the dentist’s role in thorough examination of the patient’s mouth, not just the teeth, to detect abnormalities such as ulcer or exophytic mass since ulcerative lesions are positively related to OSCC (11). This can have a significant effect in case of premalignant and malignant lesions because early detection can tremendously reduce the morbidity and mortality for the patient. The common location for OSCC is the tongue (18). Gingiva is not the predilection site for OSCC. Most OSCCs on the gingiva preferentially occur at the mandibular gingiva and more than 60% are found posterior to the premolar region (19-22) which is comparable to 74.4% of the lesions in the present study. Our study showed the mean age of gingival SCC to be 61.33 years with peak incidence in the 70-79 years age group. Gingival SCC in our study was slightly higher in woman (53.5%) which is in contrast to previous studies which showed a male predominance (5,10) but consistent with another reviewed data (23).

In the developmental disorder group, melanotic macule and nevus presenting only on the gingiva was 0.88% which is much less than the studies by Hernandez-Rios *et al*. (12) and Li *et al*. (13). Other studies (4-8,14) did not include developmental disorders in the studies.

Among the infectious diseases, fungal infection accounted for 0.25%, followed by verruca vulgaris (0.18%). Our study included only the histopathological submitted specimens, therefore the number of infectious gingival lesions diagnosed by clinical examination or other investigation such as herpetic gingivostomatitis, candidiasis and necrotizing periodontal diseases were not included in the result causing the underestimation of the prevalence of infectious gingival lesions.

Most premalignant lesions on the gingiva in this study was epithelial dysplasia (1.38%) which is comparable to study by Hernandez-Rios *et al*. (1.78%) (12). Study by Li *et al*. (13) which showed the highest incidence of OSCC on the gingiva (30.53%) also showed a high prevalence of gingival epithelial dysplasia (6.95%).

The limitations of this study are the sample size and the retrospective design of the study. If the sample size were larger and from multicenter study, it would better reflect the diverse population coverage and increase generalizability of the data. The retrospective study design was unable to identify risk factors for gingival lesions. Identifying these risk factors would have enhanced the value of this study. In addition, a number of lesions in which biopsy is not routinely performed such as gingival traumatic ulcer, necrotizing gingivitis/periodontitis, herpetic gingivostomatitis, candidiasis or other infections might lead to underreporting of the gingival lesions.

Dentists, no matter what specialties they belong to, should pay attention to not only teeth, but also to other structures such as gingiva, tongue, palate and other oral mucosa. Conditions such as premalignant lesions or oral cancers can manifest in the oral cavity even though the prevalence is relatively low compared to other gingival lesions, but they do exist. Dentists may be the first healthcare professionals to notice signs of potential problems allowing for early intervention and reducing the potential for complications or extensive treatment and their vigilance can make a significant difference in the patient outcomes. They can institute appropriate treatment, refer patients to specialists or collaborate with other healthcare professionals for further diagnosis and treatment.

## Conclusions

The most common gingival lesion in this study is PG followed by IF and POF respectively. The results of the present study are consistent with previous studies with only minor differences. The prevalence of gingival lesions from biopsied study may not reflect the true prevalence because some of the gingival lesions do not need biopsy for diagnosis. However, biopsy is still considered a gold standard for gingival lesion diagnosis because there are varieties of diseases presenting as lump, bump or other manifestations on the gingiva which cannot be diagnosed on clinical ground.

## Figures and Tables

**Table 1:** The frequency of lesions according to diseases type.

Lesions	Number	Lesions	Number	Lesions	Number
Neoplastic lesion		Non neoplastic		Non neoplastic	
Malignant		Reactive		Developmental disorders	
Squamous cell carcinoma	86	Pyogenic granuloma	484	Melanotic macule	7
Lymphoma	13	Irritation Fibroma	273	Nevus	7
Verrucous carcinoma	10	Peripheral ossifying fibroma	150	Vascular malformation	2
Melanoma	5	Gingival hyperplasia	91	Total	16
Malignant fibrous histiocytoma	2	Hyperkeratosis and acanthosis	38		
Metastatic tumor	2	Fibrous hyperplasia	19	Infection
Plasmacytoma	2	Verruciform xanthoma	12	Fungal infection	4
Angiosarcoma	1	Peripheral giant cell granuloma	11	Verruca vulgaris	3
Kaposi sarcoma	1	Plasma cell gingivitis	8	Bacterial infection	1
Liposarcoma	1	Amalgam tattoo	6	Total	8
Round cells malignant tumor	1	Papillary hyperplasia	4		
Total	124	Fibroepithelial polyp	3	Premalignant	
		Hematoma	2	Epithelial dysplasia	22
Benign		Fibrosis	1	Verrucous hyperplasia	7
Giant cell fibroma	49	Foreign body reaction	1	Carcinoma in situ	1
Squamous papilloma	34	Pseudoepitheliomatous hyperplasia	1	Total	30
Lipoma	6	Epithelial atrophy	1		
Neurofibroma	6	Total	1105		
Hemangioma	4				
Congenital epulis of newborn	3	Autoimmune diseases			
Fibrolipoma	2	Lichen planus	122		
Langerhans cell histiocytosis	2	Mucous membrane pemphigoid	46		
Benign spindle cell lesion	1	Pemphigus vulgaris	22		
Mesenchymoma	1	Bullous pemphigoid	6		
Perineuroma	1	Lupus erythematosus	1		
Total	109	Total	197		

**Table 2:** The distribution of 10 most common gingival lesions according to patients’ age, gender and location.

Category	PG	IF	POF	LP	GH	SCC	GiF	MMP	HK	SqP
Age										
0-9	12	6	1	1	2	0	5	0	0	0
10-19	81	22	23	4	21	0	6	0	0	2
20-29	84	62	44	20	14	1	6	2	2	7
30-39	91	47	41	35	13	4	9	6	3	10
40-49	72	51	18	36	15	11	8	12	6	8
50-59	71	41	8	19	14	17	5	13	12	3
60-69	43	25	12	6	8	20	9	10	9	2
70-79	23	18	3	1	3	28	0	3	4	2
80-89	5	1	1	1	1	5	0	0	2	0
90-99	2	6	23	4	0	0	1	0	0	0
Gender										
Male	138	91	46	27	31	40	17	7	25	18
Female	346	182	104	95	60	46	32	39	13	16
Location										
Maxillary gingiva	262	138	88	8	41	21	22	4	14	18
Mandibular gingiva	222	135	62	5	32	64	27	6	12	16
Maxillary and mandibular gingiva	0	0	0	109	18	1	0	36	0	0

PG: pyogenic granuloma, IF: irritation fibroma, POF: peripheral ossifying fibroma, LP: lichen planus, GH: gingival hyperplasia, SCC: squamous cell carcinoma, GiF: giant cell fibroma, MMP: mucous membrane pemphigoid, HK: hyperkeratosis and acanthosis, SqP: squamous papilloma

## Data Availability

The datasets used and/or analyzed during the current study are available from the corresponding author.
